# Comparison of Protective Immune Responses to Apicomplexan Parasites

**DOI:** 10.1155/2012/852591

**Published:** 2011-08-18

**Authors:** Sonja Frölich, Rolf Entzeroth, Michael Wallach

**Affiliations:** ^1^The ithree Institute, University of Technology Sydney, P.O. Box 123, Broadway, Sydney, NSW 2007, Australia; ^2^Institute of Zoology, Technical University Dresden, Mommsenstraße 13, 01062 Dresden, Germany

## Abstract

Members of the phylum Apicomplexa, which includes the species *Plasmodium, Eimeria*, *Toxoplasma,* and *Babesia* amongst others, are the most successful intracellular pathogens known to humankind. The widespread acquisition of antimicrobial resistance to most drugs used to date has sparked a great deal of research and commercial interest in the development of vaccines as alternative control strategies. A few antigens from the asexual and sexual stages of apicomplexan development have been identified and their genes characterised; however, the fine cellular and molecular details of the effector mechanisms crucial for parasite inhibition and stimulation of protective immunity are still not entirely understood. This paper provides an overview of what is currently known about the protective immune response against the various types of apicomplexan parasites and focuses mainly on the similarities of these pathogens and their host interaction. Finally, the evolutionary relationships of these parasites and their hosts, as well as the modulation of immune functions that are critical in determining the outcome of the infection by these pathogenic organisms, are discussed.

## 1. Introduction

Parasitic protozoans of the phylum Apicomplexa are the most prevalent and successful pathogens known to humankind. Today, half of the world's population is at risk of malaria caused by four *Plasmodium* species [1], and more than 50 billion livestock reared for food production suffer from debilitating intestinal diseases caused by many species of *Eimeria, Theileria,* and *Babesia*, amongst others [2]. *Eimeria* is the cause of coccidiosis in chickens, a parasite that infects the intestinal mucosa of the infected bird leading to severe weight loss and even death of the host. Cryptosporidiosis is caused by *Cryptosporidium* species and, like *Eimeria,* is transmitted by accidental ingestion of highly resistant and environmentally stable oocysts that contaminate the food and water. The disease is marked by self-limiting diarrhoea in immunocompetent individuals, but in immunocompromised patients, the disease can be fatal. *Babesia* is related to the malaria parasite in that it infects the reticulocytes of the infected cow and causes severe pathology and can cause death as well. *Toxoplasma* is the cause of toxoplasmosis in humans, a disease characterized by mild flu-like symptoms in healthy hosts. However, immunocompromised individuals, such as HIV/AIDS patients and organ transplant recipients, often suffer from ocular toxoplasmosis or even encephalitis. *Theileria*, an important cattle parasite transmitted by ticks, is characterized by anaemia and high mortality rate especially in pregnant cows. 

*Plasmodium* infects red blood cells and is the cause of malaria in humans as well as in several other vertebrate and bird species. Nearly one million human deaths are attributed to malaria each year, meaning that every 30 seconds a child dies of this disease in Africa. This high toll in human and animal life and wellbeing has been further exacerbated by the inappropriate use of antimicrobial compounds over the years. Thus, widespread resistance to most (if not all) drugs used to date makes control of these parasites extremely difficult [3, 4]. The novel artemisinin-based therapies are considered to be the new hope for malaria control and have proved to be successful in interrupting the maturation of the infectious stages (oocysts) in the related parasite, *Eimeria*, thereby reducing or blocking the transmission and spread of the parasite [5–7]. However, there are fears that overuse of these novel compounds will also facilitate the selection of even more potent strains. 

Over the past three decades, a number of putative protective antigens from several members of the phylum has attracted a great deal of research and commercial interest in the hope to develop vaccines and alleviate the burden on public health and world economy imposed by this class of parasites [8–17]. The first antiprotozoan subunit vaccine developed to date, CoxAbic, which contains antigens isolated from the sexual stages of development of *Eimeria* is a proof of principle for transmission-blocking immunity and an example of a strategy that has been proved successful in helping to tackle one of these important apicomplexan diseases [18–21]. 

Despite the enormous efforts to characterise the apicomplexan immunostimulatory antigens and genes encoding them, the fine cellular and molecular details of the effector mechanisms crucial for parasite inhibition and stimulation of protective immunity are still not fully understood. It is hoped that unravelling the proteomes and genome sequences of these protozoan pathogens will facilitate our understanding of the mechanisms involved in the infectious process and lead to the design of new effective control strategies [22].

Early studies concerning the developmental biology and immunology of the Apicomplexans have provided valuable insights into the immune mechanisms responsible for the inhibition of parasite growth and development and in the establishment of host resistance to infection [23–27]. Efforts by research laboratories across the globe have demonstrated that, in order to control the infection, both the innate and adaptive arms of the immune system are crucial for resistance and cross-protection [28]. This paper provides an overview of Apicomplexan biology and focuses on the protective immune response against the various types of apicomplexan parasites, from *Eimeria* to *Plasmodium* including *Toxoplasma, Cryptosporidium*, *Theileria,* and *Babesia*. It also addresses the evolutionary relationship of these parasites and their hosts and the modulation of the host immune response that are critical in determining the outcome of an infection.

## 2. Apicomplexan Life Cycle and Parasite-Host Relationship

The apicomplexan life cycle includes both asexual multiplication (schizogony, merogony) and sexual reproduction (gametogony) [25, 29, 30]. While some members of the phylum require an intermediate host and a variety of cell types to complete their developmental life cycle (i.e., *Plasmodium, Babesia, Theileria, Toxoplasma*), others lead a monoxenous life style with the asexual and sexual stages of development restricted to specific tissues of a single host (i.e., *Eimeria* and *Cryptosporidium* species). Thus, the possibility of culturing asexual stages in vitro [31], as well as the feasibility of isolating relatively large numbers of sexual forms (gametocytes), has granted *Eimeria *species a status of an attractive and a relatively simple model for investigating parasite-host interactions, as well as applying transmission-blocking immunity [32]. 

Apicomplexan parasites affect all classes of vertebrates, including fish, amphibians, reptiles, birds and mammals. The apparently long coevolutionary history of the apicomplexans means that targeting the metabolic reactions and pathways of the parasite also harms the host, making the identification of therapeutic targets extremely difficult. Recent advancements in molecular biology have shed new light into the coevolutionary history of the apicomplexa and their hosts. This is based on the finding that the branching of the evolutionary tree of at least some of these parasites coincided with the evolution of the vertebrate host [33]. In addition, it was found that several enzymes involved in a variety of metabolic pathways are highly conserved between the parasite and its host. Furthermore, comparative studies of whole genome nucleotide sequences in several members of the phylum have revealed that surface proteins, unlike the house-keeping proteins and enzymes, have evolved rapidly over the past 500 million years due to functional and immune selective pressure [33]. This is especially evident in the molecules comprising the apical complex, the invasion machinery mediating physical recognition, cytoadherence, and penetration of the host, which are parasite specific. 

The successful invasion of the host cell by the apicomplexan parasite is dependent upon the sequential secretion of proteins and other molecules from the rhoptries, micronemes, and dense granules and results in the formation of the parasitophorous vacuole (PV) (i.e., *Toxoplasma, Cryptosporidium, Plasmodium,* and *Eimeria spp*.). This in turn provides access to intracellular nutrients and protection from the host's immune system [34]. Although the PV shields the parasite from the host defences, at the same time it restricts access to nutrients in the host's cytoplasm. Thus, the Apicomplexa has adopted different tactics to circumvent this problem, including biochemical modification of the PV making it permeable to essential nutrients. In contrast, some parasites, such as *Theileria spp*., do not form the PV and proliferate freely in the cytoplasm with direct access to host nutrients [35]. 

Lateral gene transfer, best known for its role in antibiotic resistance in bacteria, has been proposed as a mechanism by which these opportunistic organisms acquire new genes that confer parasite fitness [36–38]. For example, the apical complex, actin-myosin-powered motor, evolved as a result of the nuclear transfer of genes acquired during the secondary endosymbiotic event. It is believed that origin of the apicoplast can be attributed to endosymbiotic partnership in which the plastid-containing eukaryote was engulfed by a second eukaryotic cell. In addition, sexual replication appears to be another contributing factor to the diverse functions of the apicomplexan surface proteins and the adaptations to genera-specific niches [39–41]. Therefore, it is not surprising that *Plasmodium* and *Babesia* species share evolutionarily conserved mechanisms of erythrocyte invasion [23]. Moreover, transmembrane proteins (thrombospondin-related anonymous proteins—TRAP) bridging the apical complex to the host cell in *Plasmodium, Eimeria,* and *Toxoplasma* also share a high degree of homology [34, 42–44], suggesting that Apicomplexa use the same molecular machinery to invade a wide variety of cells. 

A great deal of research has also been carried out to study the role of surface antigens in parasite growth, development, and survival. Passive transfer of polyclonal and monoclonal antibodies raised to asexual stage surface antigens of *T. gondii* are capable of conferring resistance against lethal challenge with this parasite [45, 46]. Similarly, *Eimeria* and *Plasmodium* antisporozoite and antimerozoite antibodies that recognize surface antigens, namely, glycosyl-phosphatidylinositol- (GPI-) anchored antigens in *E. tenella* (EtSAG1) and *P. falciparum* (MSP1), respectively, are able to induce a strong inhibitory response and provide protection against infection [47, 48]. In addition, the antigens found on the surface of sporozoites have been implicated in the recognition and invasion of the hepatocytes in malaria and, therefore, represent promising targets for vaccine developers [10, 42, 49, 50] (a detailed description of apicomplexan invasion and egress has been reviewed recently by Westwood and colleagues with a special emphasis on elements of the apical complex and their potential role as vaccine targets [51]). Thus, both asexual-stage surface proteins and the molecules associated with the apical complex have been proposed as potential candidates for vaccine development. 

Another conserved feature of the phylum is the transmissible, environmentally durable oocyst/cyst, the zygote stage of the Coccidia (i.e., *Toxoplasma, Eimeria, Cryptosporidium, *and* Neospora* species) [29, 52]. It is intriguing that asexual reproduction in *Eimeria* is tightly regulated, and although signals initiating the start of sexual reproduction have not yet been identified, the 2nd, 3rd, and 4th generation merozoites (depending upon which *Eimeria* species infects the host chicken) differentiate into male (micro-) and female (macro-) gametes in a synchronised manner. The trademark of apicomplexan gametogenesis is the synthesis of numerous lipid bodies and polysaccharide granules, believed to be acquired from the host cell, serving as an energy source for the developing zygote. Moreover, increased DNA synthesis and RNA transcription to produce multinucleated microgametocytes and heightened protein synthesis in the macrogametocytes to produce wall forming bodies (WFBs) are characteristics of sexual stage development at the molecular level [53–56]. *Eimeria, Cryptosporidium, *and* Toxoplasma spp*. gametogenesis is completed by the formation of environmentally resilient oocysts in the mucosae lining the gastrointestinal tract of chickens, humans, and cats, respectively. The oocysts are then excreted with the faeces where they mature (sporulate) becoming infectious. *Cryptosporidium* is slightly different from *Eimeria* and *Toxoplasma* in that the *Cryptosporidium* oocysts can also release sporozoites in the gut which are capable of infecting new epithelial cells (i.e., autoreinfection). In all three cases, transmission is primarily by the accidental ingestion of sporulated oocysts. 

Unlike the intestinal parasites described above, gametogenesis of *Plasmodium* and *Babesia spp*. is completed in the gut of their arthropod hosts. During development, after fertilization of macrogametes by the microgametes and invasion of the gut by the ookinete, the zygotes encase themselves in a protective single-layered cyst wall to form an oocyst. Flagellated sporozoites then exit the oocyst and migrate from the midgut to the salivary gland of their arthropod host. From there, they are injected into the blood and subcutaneous tissue of the next vertebrate host the arthropod bites.

## 3. Naturally Acquired Immunity to Apicomplexan Parasites Is Exclusively Dependent upon Cell-Mediated Immunity

Studies to elucidate the mechanism(s) of the protective immune response to apicomplexan parasites have been limited mainly due to the lack of being able to carry out such studies in the definitive host such as cattle or human beings. Thus, much of our understanding of the protective immune response to apicomplexan infections has been derived from murine models which can be genetically modified [26, 57]. Generally speaking, parasite replication in the host eventually leads to host cell lysis and parasite egress. It is now widely accepted that cells of the innate immune system and the molecules they produce and/or secrete are important controlling factors of parasite infectivity and in limiting the extent of parasitemia [58–61]. It is not clear exactly how these molecules, particularly in the case of parasites infecting host erythrocytes (i.e., *Plasmodium* and *Babesia spp*.), interfere with parasite development. What does appear to be the case is that this inhibition is accomplished by the production of gamma interferon (IFN-*γ*),by natural killer cells (NK), and tumour necrosis factor alpha (TNF-*α*), nitric oxide (NO) and reactive oxygen species (ROS) by macrophages [62–64]. Despite the fact that the mechanisms by which IFN-*γ* mediates protection are not completely understood, studies using IFN-*γ* and iNOS knock-out mice infected with *T. gondii *indicated that activation of p47 guanosine triphosphatases (GTPases) leads to degradation of the PV in infected cells [65]. 

While the immune response in healthy hosts is often but not always able to control parasite replication and limit the disease, immunocompromised individuals fail to stop parasite growth, and clinical disease develops in nearly all cases. This is especially evident in toxoplasmosis where immunocompetent hosts control parasite replication causing tachyzoites (the rapidly replicating asexual stages) to migrate to muscle and brain tissues where they differentiate into bradyzoite cysts (the slow-replicating form) and persist throughout the host's life. Although the tissue cysts can become reactivated periodically, most healthy hosts never develop clinical disease. In contrast, immunocompromised patients, such as those suffering from AIDS, remain chronically infected, whereby reactivation of the tissue cysts can lead to toxoplasmic encephalitis with severe pathological consequences. 

The ability to clear acute and chronic infections with the Apicomplexa seems to correlate with host CD4+ T-cell levels [62, 66, 67]. Thus, studies involving athymic animals have shown that T cells play a crucial role in the infectious process [62, 68]. 

The population of T cells coexpressing *αβ* markers appears to be important for host defence against apicomplexan infections [69, 70]. Thus, it was demonstrated that TCR*α*-deficient mice developed severe disease when compared to controls [70]. Furthermore, mice lacking major histocompatibility complex class II expression (i.e., CD4+ T-cell deficient) appeared more susceptible to apicomplexan infections [62, 67].

CD8+ T cells also appear to play a role in parasite growth and dissemination because they provide sporozoite transport from their initial infection site, as is the case for *Eimeria* and *Toxoplasma* infections [69, 71]. During the course of primary infection with *Eimeria*, CD8+ T cells appear to play a role in parasite growth and dissemination because they provide sporozoite transport from their initial infection site to the crypt cells. On the other hand, reports have also shown that increased numbers of CD8+ T cells in the crypt epithelium act as cytotoxic killer cells facilitating the clearance of the parasite-infected cells [72, 73]. Furthermore, studies in infected chickens have shown that the contribution of both CD4+ and CD8+ T-cell populations differs according to the infective species used [70]. Nevertheless, during avian coccidiosis and babesiosis, CD4+ T-cell subsets were found to be elevated in animals following challenge infections [67, 70, 72]. An intriguing question arises from all of these observations: why are some species or strains of parasites extremely “immunogenic” and induce protective immunity, while others seem to be invisible to the immune system? 

Studies on naturally acquired immunity to malaria have shown that adequate protective immunity to *P. falciparum*, the etiological agent of the most severe malaria in humans, usually required repeated infections. Thus, protection against this particular strain appeared to be acquired more slowly than against the less pathogenic *P. vivax* or *P. malariae* [28]. Moreover, numerous studies have shown that immunity appeared to be species specific and did not confer protection against challenge with heterologous species. However, it was reported that heavy exposure to parasites induces the development of antigenic memory [74]. Although the molecular and cellular mechanisms driving the onset of protective host immunity against malaria or any other pathogenic protozoan are not entirely understood, it is believed that susceptibility to infection is driven by extrinsic factors such as antigenic variation and also by intrinsically inappropriate immune responses [75]. This is particularly evident in *Theileria* infections of immunocompromised cattle, which often results in the death of the host animal. It is theorised that the ability of *Theileria* to interfere with the host's apoptotic pathways is the crucial factor contributing to mortality [35, 76]. Once inside the host cell, *Theileria* resides free in the cytoplasm and induces uncontrolled proliferation of the infected cell. Some have even compared *Theileria* infections with cancer development and metastasis. Thus, it appears that during *Theileria* infection the immune system fails to control this proliferation in time, in turn resulting in potent, nonspecific lysis of both infected and noninfected cells. 

Studies using immunocompetent animals have shown that, in addition to innate host resistance, IFN-*γ* plays a key role in the development of adaptive immunity and clearing of Apicomplexa infections. For example, *Cryptosporidium*-infected mice, with faulty IFN-*γ* gene expression, suffered from severe mucosal destruction, and as a result they also secreted more oocysts [60, 77]. Furthermore, mopping-up the secreted IFN-*γ* by antibodies in immunocompromised animals seemed to worsen *C. parvum* infection. In addition, it is now widely accepted that IL-12, known to increase host IFN-*γ* production, can reduce the severity and even prevent infection by apicomplexan parasites [63]. Although believed to be mediated by an IFN-*γ*-dependent mechanism, the pathways or downstream molecules crucial in this process have not been well defined to date.

## 4. Masters of Disguise

Although viruses, which entirely depend on the host machinery for replication and assembly of new viral particles, are the experts in host cell manipulation, the Apicomplexa are considered to be the masters of disguise. This is because they have evolved to evade the host immune system to aid in their own survival. Antigenic variation has been proposed as a key factor in this process. Unlike allelic polymorphism, which results in different phenotypes or so-called parasite strains [78], antigenic variation is the tightly regulated expression of different genes of a clonal population of parasites over the natural course of infection [75]. Antigenic variation amongst malarial and *Babesia* parasites is a prime example of sophistication apicomplexans employed to avoid antibody-mediated inhibition [79–81]. *P. falciparum* achieves this by secreting a single type of a variant molecule (parasite-derived erythrocyte membrane protein 1 - PfEMP1) on the surface of the infected erythrocyte at any one time. The PfEMP1 surface proteins are encoded by a family of genes, called *var* genes, and each individual parasite expresses only a single *var* gene, keeping all other members of *var* gene family in a transcriptionally silent state [82–84]. This strategy in turn induces adhesion of the parasite-infected erythrocytes to the blood vessels to avoid reaching the spleen, whose main function is to rid the body of damaged and/or infected blood cells. Similarly, sequestration of *Babesia*-infected erythrocytes in the microvasculature enables the *Babesia* to persist within the host maximizing its chances of transmission [81]. This cytoadherence in *Babesia* is mediated by constant gene conversion of *ves* family genes encoding the variant erythrocyte surface antigen 1 (VESA1) [85]. 

A puzzling question arises from these observations: if infected erythrocytes pass through the body unchecked since they lack major histocompatibility complex (MHC) expression, overwhelming proliferation of the parasites may cause premature death of the host prior to successful transmission to an arthropod vector? Interestingly, in spite of the fact that malaria parasites sequentially express variant surface molecules exposing the immunodominant antigens to the host immune defences, infection is actually prolonged. Thus, the parasite must undergo antigenic variation and rates of growth that enable the host to control infection while allowing for transmission of the parasite prior to its death. This mechanism to prolong infection was also evident in merozoites of *Babesia* species. Due to coating of the merozoite surface with glycosyl-phosphatidyl-anchored proteins crucial for initial attachment to the host erythrocyte surface, they are targeted by host-protective antibodies [86]. These surface-anchored proteins (Variable Merozoite Surface Antigens-VMSA) exhibit varying degrees of intra-species antigenic polymorphisms allowing these parasites to evade the host immune system at the population level [75]. Nevertheless, studies involving African children have shown that variant specific immunity, namely, secretion of IgG antibodies directed against *P. falciparum* variant surface antigens (VSA), has been correlated with protection from clinical malaria in Ghana, Kenya, and Tanzania [79, 87, 88]. Thus, VSA antigens have been proposed as excellent candidates for malaria and *Babesia* vaccine development.

## 5. Humoral Immunity Protects against Challenge Infections

Although B cells have been regarded as minor contributors to protective immunity and resistance to primary infections with Apicomplexa, numerous studies have shown that hosts infected with these parasites are capable of producing protective, parasite-specific immunoglobulins (Ig) of all major classes after an episode of infection and recovery [57, 89–92]. Thus, early work by Rose and colleagues has shown that humoral antibodies, induced by live *Eimeria* infection, can provide excellent passive protection against challenge infections with the same parasite [93, 94]. Likewise, studies on mice infected with *T. gondii* have shown that intestinal IgA antibodies to major surface protein SAG-1 (P30) were produced after peroral infection and found to inhibit infection of murine enterocytes by directly blocking the parasite entry [91]. In addition, Precigout et al. have demonstrated an inhibitory effect of antibodies directed against a 17-kDa merozoites membrane protein on *B. divergens* parasite growth [92]. Furthermore, studies on invasion of red blood cells by *P. falciparum* merozoites have revealed that since RBCs do not express the MHC complex, parasite killing by T lymphocytes is not important. Instead, antibodies specific to merozoite surface molecules (MSP-1) and proteins externalised from the apical complex play a major role in immunity to asexual blood stages [95]. 

The *Plasmodium* merozoite surface protein 1 (MSP-1) is a 200 kDa multicomponent precursor complex derived by proteolytic processing during erythrocyte invasion. The 42 kDa C-terminal component is cleaved (i.e., secondary processing) to produce soluble 33 kDa and 19 kDa fragments that remain on the merozoites surface [96]. Studies have shown that anti-merozoite antibodies are capable of neutralizing parasites by Fc-dependent mechanisms involving macrophages, thus reducing the parasitemia and clinical disease [87, 97, 98]. In addition, a number of recent studies have shown that children naturally infected with malaria secrete anti-MSP-1 antibodies (MSP-1_19_ mAb) that block the binding of *Plasmodium* merozoites to the surface of the red blood cells and also inhibit secondary processing of MSP-1. In addition, studies investigating the protective properties of maternally derived IgG and IgM antibodies to the 19 kDa domain of MSP-1 of *P. falciparum* have shown that mothers who have tested positive for anti-MSP-1 (19 kDa fragment) IgG antibodies conferred protection against placental infection and infection in their infants [99]. 

It has been shown that, in *babesiosis* infection, IgG antibodies produced as a result of live infection can prevent infection of erythrocytes by binding and neutralizing sporozoites before they invade their target cells. Similar observations were reported in chickens where antisporozoite antibodies specific to glycosyl-phosphatidylinositol-anchored *E. tenella* surface antigen 1 (EtSAG1) appeared to inhibit parasite binding and invasion of the host cell [24]. However, it seems that the protective role of these antibodies is limited since it can only neutralize sporozoites from the time the parasites egress and the time they gain access to new cells. Thus, it is hoped that genome-wide fingerprinting techniques [100] will aid in the identification of additional immunoprotective antigens that can be used in combination to induce the maximal inhibitory humoral immune response. 

In addition to antigen-specific polyclonal and monoclonal antibodies capable of inhibiting asexual stages, antibodies raised to antigens localized exclusively to gametocyte/zygote stages were also found to be highly immunogenic and capable of providing passive protection in vivo [20, 101, 102]. Early experiments involving immunisation with purified sexual-stage gametes of *P. gallinaceum* in chickens showed that effective transmission-blocking immunity can be achieved by reducing the infectivity of gametocytes and oocyst development [103, 104]. Thus, Pfs25 and Pvs25 proteins expressed on the surface of ookinetes in the mosquito stage of *P. falciparum* and *P. vivax* have been used extensively as candidates for malaria transmission-blocking vaccines, since lowering the density of circulating parasites would not produce sterilizing immunity, instead it would allow individuals to develop long-lasting, naturally acquired immunity to malaria [12, 105, 106]. 

**Figure 1:**
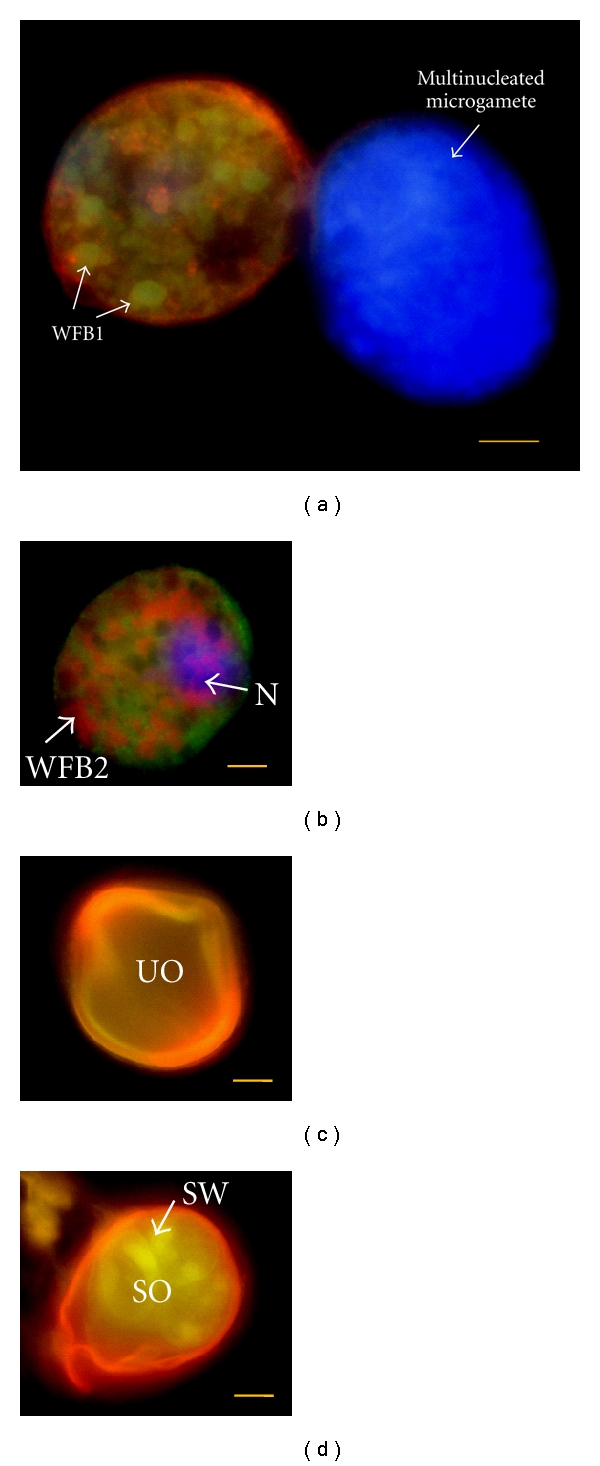
Developing and mature micro- and macrogametes and oocysts harvested from the infected chicken intestine 134 h post infection (p.i.) and double-labelled with a monoclonal antibody raised to antigens confound to WFB1s (E1D8) [107], and a polyclonal affinity purified gametocyte antigens Emgam56, Emgam82, and Emgam230, amongst other molecules (anti-APGA) specific to molecules contained within the wall-forming bodies (1 and 2) [53] and visualised with fluorescein isothiocyanate (green) or revealed with rhodamine-conjugated goat anti-mouse IgG secondary antibody (red). Counterstained with DAPI. Abbreviations: N, nucleus; SO, sporulated oocysts; SW, sporocyst wall; US, unsporulated oocyst; WFB1, wall-forming body type 1; WFB2, wall-forming body type 2. Bars represent 5 *μ*m.

Work by Wallach and coworkers, aimed at applying transmission-blocking immunity to control infections caused by *Eimeria,* hypothesised that antibodies raised against the gametocyte/zygote stages of development can act to inhibit oocyst development and thereby provide a block in parasite transmission (see Figure 1). A method was developed for purifying *E. maxima* gametocytes from the infected chicken gut mucosa and immunodominant gametocyte antigens, namely, Emgam56, Emgam82, and Emgam230 localized to the WFBs and the oocyst wall of the maturing zygote, were extracted [53, 102, 108]. Passive immunisation experiments showed that there was a good correlation between the intensity of IgG and IgM antibodies binding to gametocyte antigens by Western and ELISA with the ability of those sera to provide passive protection in vivo [109]. The mechanisms by which these antibodies inhibit oocyst maturation are still obscure; however, it is hypothesised that antibodies raised to the immunodominant antigens retard zygote development by interfering with the processing of wall proteins or the wall-hardening processes [53, 110]. In addition, a protective monoclonal antibody raised against Emgam56 localised to the WFB2 (1E11-11), as well as the inner layer of the oocyst wall, was also found to react strongly with the Stieda body of the sporulated oocysts (M. Wallach, unpublished data). Similar results were reported by Krücken et al. using a monoclonal antibody E2E5 raised to WFB2s of *E. tenella* [101]. The in vitro excystation inhibition assay showed that the antibody E2E5 can significantly interfere with parasite development by impairing sporozoite excystation. It is tempting to speculate that the 1E11-11 monoclonal antibody inhibits or blocks excystation of the sporocyst in a similar manner, thereby reducing the number of infectious sporozoites released in the intestine of infected birds allowing them to develop protective immunity induced by exposure to low doses of parasites. 

Jenkins and colleagues have shown that ruminants immunized with a DNA vaccine expressing a gene isolated from *C. parvum* encoding a sporozoite antigen (CP 15/60) were capable of inducing antigen-specific antibodies [111, 112]. In that study, it was found that using various routes of vaccination resulted in differing antibody responses and titres. The authors, therefore, suggested that the route of antigen delivery of any protozoan vaccine requires careful formulation and optimisation of delivery systems.

Finally, in studies carried out by Wallach and co-workers on *Eimeria* [109], it was found that in order to achieve protective immunity using parasite extracts requires the inclusion of the correct antigens and exclusion of the irrelevant ones. Their results indicated that while some parasite-specific antigens induce protective immunity, others actually induce an exacerbation of the infection. Therefore, in the design of any parasitic vaccine, it is crucial that the combination of various antigens maximizes their inhibitory effect on parasite growth and development.

## 6. The Apicomplexa Are the Manipulators of Host Defence Mechanisms

One of the main defence mechanisms employed by host cells is programmed cell death (apoptosis) ensuring regulated removal of damaged and infected cells [113]. But because the survival and development of intracellular apicomplexan parasites is dependent upon the continuous supply of host cell nutrients and protection from immune attack, the parasites have adapted to extend the life of the infected cells by inhibiting the host cell apoptotic machinery through interference with the intracellular signalling molecules, notably phosphatidylinositol 3-kinase (PI3-K). PI3-K is involved in a variety of functions including cell growth, proliferation, and intracellular trafficking, amongst others [61, 114–117]. *P. falciparum* is a good example of how parasite secreted proteins prevent host cell death to ensure its own development and survival. Sporozoites of *Plasmodium* species are stealthy invaders that first travel to the liver (hepatocyte) cells, where the growth and development of the daughter cells, hepatic merozoites, takes place. Recent results have shown that prior to the establishment of the PV, sporozoites of *P. falciparum *transmigrate through a number of hepatocytes before they anchor to and invade the suitable cell via exocytosis of proteins contained within the apical complex. It has been shown that the thrombospondin-related adhesive-protein- (TRAP-) like protein plays a role in this process. Additionally, the wounding of the hepatocyte induced by invading sporozoites releases growth factors which in turn appear to inhibit PI3-K and block the signalling pathways destined for apoptosis. Leirião and colleagues have shown that once the parasite is established in the hepatocyte, it secretes HGF/MET signalling molecules into the host cell cytoplasm, thereby conferring resistance to apoptosis to ensure survival and maturation of the daughter cells [117]. However, which signalling upstream of PI3-K occurs during *Plasmodium* infection is yet to be determined. Interestingly, upon maturation of merozoites, *Plasmodium* seems to be able to induce host cell death to liberate the motile progeny. Strum et al. have shown that this process involves cysteine proteases [49, 50]. Moreover, similar mechanisms were found to play a role in release of sporozoites from the oocysts [118]. Although work is ongoing to try and elucidate the mechanisms involved in these processes, it appears that the Apicomplexa have learned to inhibit host cell death during parasite development and subsequently activate it, liberating thousands of new progeny. 

*T. gondii* has also evolved a broad spectrum of adaptations to challenges presented by its life style [119]. Chronic toxoplasmosis is the trademark of the parasite's success and is induced by the slow-replicating bradyzoites safely tucked away in the remodelled PV, the tissue cyst. Like *Plasmodium*, *T. gondii* modulates host cell apoptosis by both inhibiting and triggering the programmed cell death [120]. Chen et al. have shown that Fas/FasL ligand-dependent mechanisms mediate the inflammatory responses induced by the apicomplexan infection [115, 121]. But the parasites have evolved to neutralize granzyme/perforin-mediated killing of infected T cells and natural killer cells (NK) by modifying transcription and posttranscriptional modification of IFN-*γ*-regulated genes, the major mediators of resistance to *T. gondii* infections. Likewise, del Cacho et al. have demonstrated that *E. tenella* and *E. necatrix* second-generation schizonts first induce NF-*κβ* activation to protect the transformed cells from apoptosis, allowing the schizonts to mature and later cause NF-*κβ* inhibition to trigger host cell apoptosis to facilitate the release of merozoites [7]. 

The Apicomplexa have evolved to live in synergy with their infected hosts because they completely depend on it for survival; however, some apicomplexan infections induce a great deal of immunopathology and can lead to host cell death. For example, *Eimeria* and *Cyclospora* both interfere with the absorption of nutrients across the intestinal mucosa and can cause death due to malaise, diarrhoea, and dehydration. Because apicomplexans increase in numbers while, in their hosts, the severity of infection is proportional to the parasite density—the smaller the number, the greater the chance of asymptomatic infection and the greater the chances of the parasite survival. However, the immunological defence of a host can also cause extensive tissue damage and clinical symptoms. Patients with cerebral malaria usually have elevated levels of tumour necrosis factor alpha (TNF-*α*) and IgE considered to be responsible for fever and tissue lesions to an extent where vital functions of the host fail leading to a coma [122].

## 7. Are We Losing the Battle against Apicomplexan Parasites?

Despite a great deal of effort and technological advancements in biotechnology, molecular biology, genetics, immunology, and vaccinology, there are no vaccines for humans against malaria and toxoplasmosis at the present time, and it seems that we are losing the battle in the fight against pathogenic protozoans. The current failure to develop a practical vaccine may well be attributed to our inadequate understanding of the mechanisms underlying (1) the naturally acquired immunity against apicomplexans, (2) acquired parasite resistance to most (if not all) antimicrobial compounds used to date, and (3) in the case of arthropod transmitted protozoans, failure to implement adequate vector control programs in tropical and subtropical regions. 

The life cycles of the Apicomplexa are complex, thus, it is hoped that a multivalent, multistage vaccine will alleviate the problems caused by these pathogenic protozoans. Although this approach has attracted a great deal of commercial and research interest, the critical issues to be addressed include the identification of stage-specific antigens capable of inducing protective immunity and the delivery methods in a form that will stimulate an adequate protective immune response. The main impediment in the search and selection for immunostimulatory antigens is the lack of in vitro assays to analyse and predict immune responses. The transmission blocking assays, relying on counting the number of oocysts produced, and the inhibition of sporozoite invasion assays have both been used extensively to evaluate parasite inhibition induced by neutralizing antibodies. 

Although in vivo experimentation is extremely difficult for malaria, other model systems can be used to dissect the fine details and the effect of neutralizing antibodies. It is very possible that, in the development of an antiprotozoan vaccine capable of inducing only partial immunity, resistant mutants would be selected that are even more pathogenic than existing strains. In the malaria scenario, this could be catastrophic since the parasite would undergo recycling and be transmitted throughout the community leading to an increase in morbidity and mortality. It is, therefore, of great hope that in the battle against these pathogenic protozoan parasites, including *Plasmodium*, *Cryptosporidium,* and *Toxoplasma*, the completion of their genomes and proteomes may provide information needed to design vaccines, assess the effects of immunization on parasite pathogenicity and the selection of unwanted mutants, and in the final analysis control the diseases caused by this class of parasites.
